# Prevalence of anxiety and depression and their associated risk factors throughout pregnancy and postpartum: a prospective cross-sectional descriptive multicentred study

**DOI:** 10.1186/s12884-024-06695-6

**Published:** 2024-07-25

**Authors:** Marta Jimènez-Barragan, Gemma Falguera-Puig, Jorge Juan Curto-Garcia, Olga Monistrol, Engracia Coll-Navarro, Mercè Tarragó-Grima, Olga Ezquerro-Rodriguez, Anna Carmona Ruiz, Laura Codina-Capella, Xavier Urquizu, Amparo del Pino Gutierrez

**Affiliations:** 1 Universitat de Barcelona, Fundació Assistencial Mútua Terrassa, (Terrassa), Research Group on Sexual and Reproductive Healthcare (GRASSIR), (2021-sgr-01489), Barcelona, 08221 Spain; 2 Atenció a la Salut Sexual i Reproductiva Metropolitana Nord, Direcció d’Atenció Primària Metropolitana Nord, Institut Català de la Salut, Barcelona, Spain; 3 AstraZeneca F, Barcelona, Spain; 4 Faculty of Nursing and Physiotherapy, University of Lleida, Iguada, Spain; 5 Head of Midwifery, Fundació Assistencial Mútua Terrassa, Terrassa, Spain; 6 Midwife, Sexual and Reproductive Health Clinic (ASSIR) CAP Rambla Terrassa, Mollet, Spain; 7 Department of Obstetrics and Gynaecology, Fundació Sanitària Mollet, Mollet, Spain; 8 Department of Obstetrics and Gynaecology, Fundació Assistencial Mútua Terrassa, Terrassa, Spain; 9 Departament de Salut Pública, Facultat de Medicina i Ciències de la Salut, Salut Mental i Materno-infantil, Universitat de Barcelona, Barcelona, Spain; 10 Research Group on Sexual and Reproductive Healthcare (GRASSIR), (2021-sgr-01489), Barcelona, 08007 Spain; 11 Institut Universitari d’Investigació en Atenció Primària Jordi Gol (IDIAP Jordi Gol), Barcelona, Spain; 12 Ciber Fisiopatología Obesidad y Nutrición (CIBERObn), Instituto Salud Carlos III, Madrid, Spain; 13 ASSIR Fundació Assistencial Mútua Terrassa, Universitat de Barcelona, Plaça Dr. Robert 5, Barcelona, 08221 Spain

**Keywords:** Anxiety, Depression, Pregnancy, Screening, Risk factors, Midwifery

## Abstract

**Objective:**

To assess the prevalence of anxiety and depression and their associated risk factors throughout the pregnancy and postpartum process using a new screening for the early detection of mental health problems.

**Design:**

A prospective cross-sectional descriptive multicentred study. Participants were consecutively enrolled at ≥ 12 weeks’ gestation and followed at three different time points: at 12–14 weeks of pregnancy, at 29–30 weeks of pregnancy, and 4–6 weeks postpartum. All women completed a mental screening at week 12–14 of pregnancy consisting of two questions from the Generalised Anxiety Disorder Scale (GAD-2) and the two Whooley questions. If this screening was positive, the woman completed the Edinburgh Postnatal Depression Scale (EPDS).

**Setting:**

Seven primary care centres coordinated by a Gynaecology and Obstetrics Department in the city of Terrassa (Barcelona) in northern Spain.

**Participants:**

Pregnant women (*N* = 335, age 18–45 years), in their first trimester of pregnancy, and receiving prenatal care in the public health system between July 2018 and July 2020.

**Findings:**

The most relevant factors associated with positive screening for antenatal depression or anxiety during pregnancy, that appear after the first trimester of pregnancy, are systematically repeated throughout the pregnancy, and are maintained in the postpartum period were: a history of previous depression, previous anxiety, abuse, and marital problems. In weeks 12–14 early risk factors for positive depression and anxiety screening and positive EPDS were: age, smoking, educational level, employment status, previous psychological/psychiatric history and treatment, suicide in the family environment, voluntary termination of pregnancy and current planned pregnancy, living with a partner and partner’s income. In weeks 29–30 risk factors were: being a skilled worker, a history of previous depression or anxiety, and marital problems. In weeks 4–6 postpartum, risk factors were: age, a history of previous depression or anxiety or psychological/psychiatric treatment, type of treatment, having been mistreated, and marital problems.

**Conclusions:**

Early screening for anxiety and depression in pregnancy may enable the creation of more effective healthcare pathways, by acting long before mental health problems in pregnant women worsen or by preventing their onset. Assessment of anxiety and depression symptoms before and after childbirth and emotional support needs to be incorporated into routine practice.

**Supplementary Information:**

The online version contains supplementary material available at 10.1186/s12884-024-06695-6.

## Introduction

Motherhood is a normal life process characterized not only by physiological and biological changes in the mother’s body, but also by a psychological adjustment to the new reality of pregnancy, childbirth, and the future baby [1]. Non-psychotic perinatal mental disorders (PMDs) refer to common mental health conditions (i.e., anxiety, depression) that occur during pregnancy or in the first year after birth. Most of these PMDs have a non-negligible prevalence worldwide. Different countries have characterized the prevalence of anxiety and depression in their pregnant and postpartum populations. In western countries the prevalence of antenatal and postnatal anxiety and depression varies: e.g., Canada, 16% and 17%, 4% and 5%, respectively [2]; Italy, 21% and 25%, 22% and 13%, respectively [3]; Spain, 4% and 15% in the postpartum period [4]. Even in certain subpopulations (low and lower-middle-income countries), the average prevalence of PMDs (mainly anxiety and depression) is about 16% prenatally and 20% postnatally [5], and between 10% and 13%, respectively, in high-income countries [6, 7]. A 2015 meta-analysis showed a high variation in the percentage of mothers with perinatal anxiety, from 3 to 39% [8]. Against this background, estimates of anxiety and depression vary considerably by the population studied, and even the evidence is inconclusive as to whether the prevalence among pregnant women differs from that of non-pregnant populations [9].

Furthermore, the negative relationship that anxiety and depression during pregnancy can have for both mother and baby, due to the increase in the hormone cortisol has been demonstrated [10, 11]. In addition, anxiety processes in pregnant and postpartum mothers lead to increased smoking and alcohol consumption [12–14], which in turn will have a clear impact on the foetus and on breastfeeding infants [15]. Anxiety during pregnancy has also been associated with a higher number of follow-up visits [16], a higher likelihood of caesarean section and requesting an elective caesarean Sect. [17]. Similarly, those who expressed distress during childbearing were more likely to experience disaffection towards their babies and postpartum depression (PPD) [2, 18]. These factors are of relevance as they negatively influence pregnancy, childbirth, and the postpartum period, and affect women’s coping, with high health, economic and social costs across cultures [19]. Some studies link maternal anxiety to adverse neurodevelopmental outcomes in the baby, including cognitive, language, emotional, and behavioural problems [11, 20]. Prenatal anxiety has also been associated with low weight for gestational age, pre-term birth, endocrine and inflammatory changes in infants, and even foetal physiological changes (heart rate abnormalities), among other effects [20–23].

However, despite the health and social impact of PMDs [24], even with a worrying associated childhood morbidity [25], there is little research on PMDs, with prevalence not always concordant and conclusive, and relatively little funding for research and specialized services and public health interventions to improve detection and management of such PMDs in pregnant women.

It is clear from the above studies that there is no common systematization in determining the existence of PMDs and associated factors, partly due to the use of different instruments to measure some of the mental conditions. In this sense, the significant heterogeneity in the methodology of the studies may explain the discrepancies between them. There are even studies that try to assess which instruments seem to be the most accurate for assessing some of the PMDs in women, based on their co-morbidity [2, 25] during pregnancy or postpartum [26].

The introduction of preventive perinatal mental health screening is a quality standard in pregnancy and postpartum management [27], as assessment, detection, management, and treatment of mental health problems that women may experience during pregnancy and postpartum are essential to ensure optimum health outcomes for both mother and baby, as well as for the rest of the family. In Catalonia, the Protocol for Pregnancy Control, in its latest revision of 2018, introduces for the first time, and in a systematic way, a universal screening in perinatal mental health [1]. This preventive intervention improves the detection of potential mental health problems that may occur in women during pregnancy and puerperium. To correctly detect and assess these problems in pregnant women, this new protocol recommends a good anamnesis, as well as the initial identification of symptoms of depression and/or anxiety through the Whooley Questions [28], the Generalised Anxiety Disorder Scale (GAD-2) [29–31], and the Edinburgh Postpartum Depression Scale (EPDS) [32], and using a timetable based on different pregnancy and postpartum periods.

The aim of this study is to determine the pre- and post-natal evolution of anxiety and depression symptoms and associated factors at 12–14 weeks and at 30 weeks of gestation, and during the puerperium (at 4–6 weeks postpartum), in a large population of women attending our services by using a standardised protocol resulting from the consensus of a large group of professionals and scientific societies in the obstetric field [1]. This protocol introduces a new screening for the early detection, by midwives, of mental health problems that may affect pregnant women, who are particularly vulnerable at this time of life. This will help to confirm the usefulness of this pre- and post-natal screening performed by a midwife, as a first step. We believe that our research to accurately determine the prevalence, understand the course, and identify risk factors and outcomes related to anxiety and depression in pregnant women can have a real impact on effective treatments for anxiety and mood disorders in pregnancy.

## Materials and methods

This is a prospective descriptive multicentre study (7 primary care centres involved) co-ordinated by the Gynaecology and Obstetrics Department in Mutua Terrassa (Barcelona, Spain) between July 2018 and July 2020 and including 335 cognitively normal pregnant women (mean age [range] 32.0 [18.0–45.0] years) who regularly come to the Sexual and Reproductive Health Care (ASSIR) unit of Mutua Terrassa to control their pregnancy. Participants were consecutively included at ≥ 12 weeks gestation.

The inclusion criteria involved: (1) women aged ≥ 18 years; (2) women controlled at the ASSIR unit of Mutua Terrassa; and (3) verbal and written comprehension of instructions and the assessment. The exclusion criteria involved: (1) any condition preventing demographic or mental health assessment, and (2) women with a diagnosed psychiatric pathology who were being monitored by the mental health team.

**Fig. 1:**
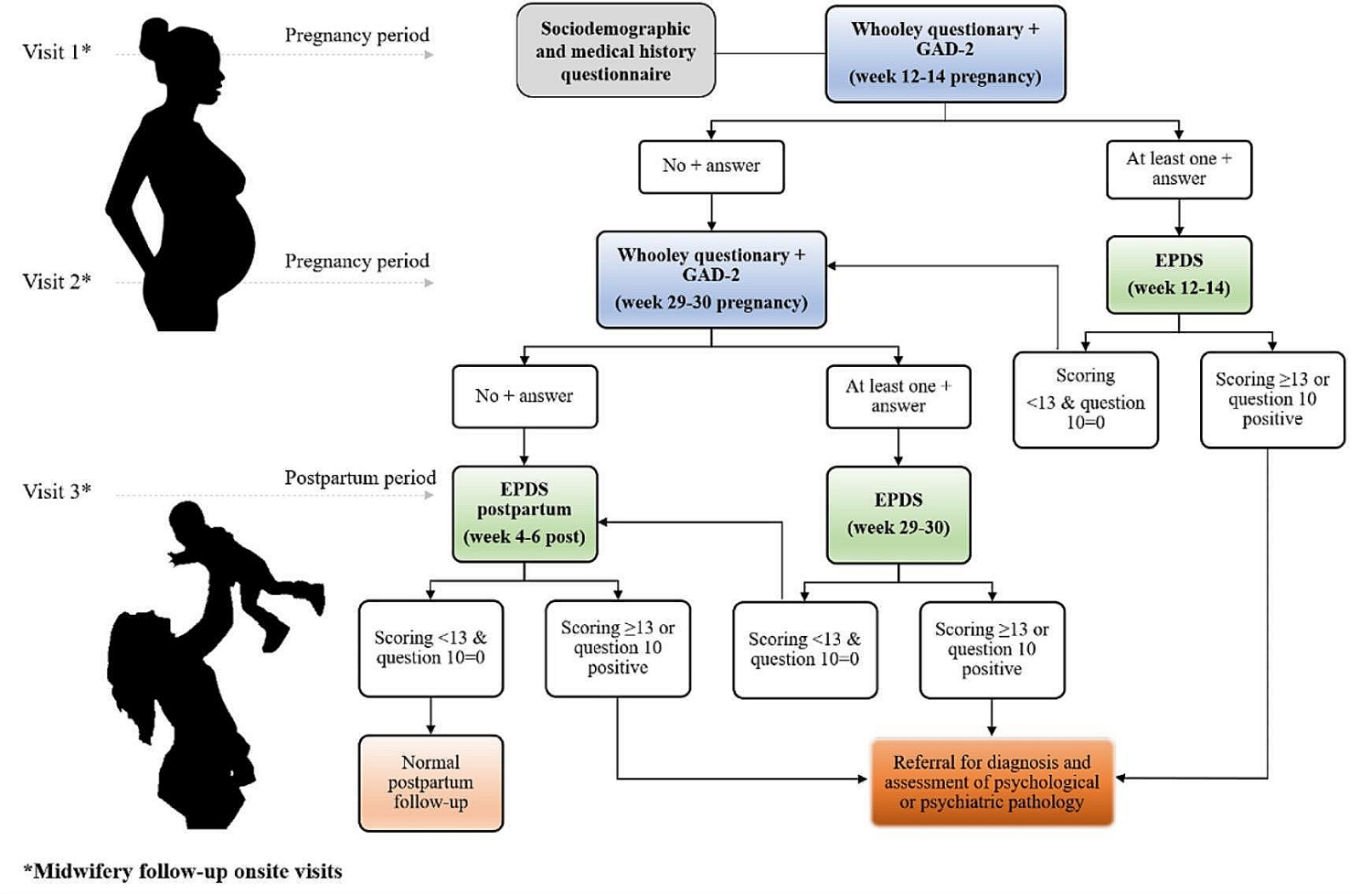
Screening for anxiety and depression symptoms in pregnant women and during the early postpartum period by the Protocol for Pregnancy Control [1]

Following a standardised protocol created by obstetric experts [1], all participants underwent an initial demographic and medical interview and a mental health screening by midwifery visits to detect possible anxiety and depression-related symptomatology (see below) at three different time points: at week 12–14 of pregnancy (Visit 1), at week 29–30 of pregnancy (Visit 2) and at week 4–6 of postpartum (Visit 3) (see Fig. 1). Mental health screening was conducted at 12–14 weeks of gestation, at 29–30 weeks of gestation, and during the postpartum visit, as described in the Pregnancy Monitoring Protocol in Catalonia (Spain), which is agreed upon by the group of experts from the Department of Health and the scientific societies of Catalonia [1].

The study was approved by the Clinical Research Ethics Committee (CEIC) of Mutua Terrassa (Spain) (# approval: B1803) and was conducted in accordance with the principles of the Declaration of Helsinki (World Medical Association Declaration of Helsinki, 1997) and its later amendments (revised in 2013). Informed written consent was obtained from all the participants. Informed consent to participate was obtained for illiterate participants from their legal guardians.

### Sociodemographic and clinical screening for mental health disorders

At the 12-14-week gestational visit, the women were enrolled in the study, completed the sociodemographic and medical history questionnaire, and underwent mental health screening consisting of the two GAD-2 questions and the two Whooley questions. If one of the four questions was positive, the EPDS was performed. In the 30th week of pregnancy, the mental health screening was performed again, and in the 4th-6th week postpartum the EPDS was performed (see Fig. 1).

Sociodemographic characteristics, current obstetric history, current mental health history, and family history information were collected at 12–14 weeks of pregnancy.

Following the guidelines of the National Institute of Clinical Excellence (NICE) [27] and the Pregnancy Control Protocol in Catalonia [1], we performed a perinatal mental screening using different questions and instruments to identify symptoms of depression and/or anxiety at 12–14 weeks of pregnancy. Specifically, the Whooley questionnaire for depression and two anxiety-related questions from the GAD-2 were used first. Based on the results of these two instruments, the EPDS was passed (Fig. 1). The specific tests for the clinical screening can be found in the Supplementary Material.

### Statistical analyses

Descriptive statistics were calculated for each sociodemographic, clinical, and obstetric variable, using median and range for quantitative variables, and absolute (n) and relative (%) frequencies for categorical variables. The McNemar test was used to compare the results of the initial screening (Whooley and GAD-2) between visits. Complementary exploratory analyses were performed at each visit to assess potential risk factors associated with a positive screening and EPDS result. Independent t-test was used for comparisons between groups in continuous variables and Mann Whitney U test was used when the variables did not display a normal distribution and the chi-square test or, when appropriate, Fisher’s exact test was used in the case of qualitative variables. All analyses were conducted using IBM SPSS Statistics for Windows, version 26.0 (Armonk, NY: IBM Corp. Released 2019). As this was a preliminary study, α was set at a p-value < 0.05, without correction for multiple comparisons.

## Results

### Sociodemographic, clinical, and obstetric variables

**Fig. 2:**
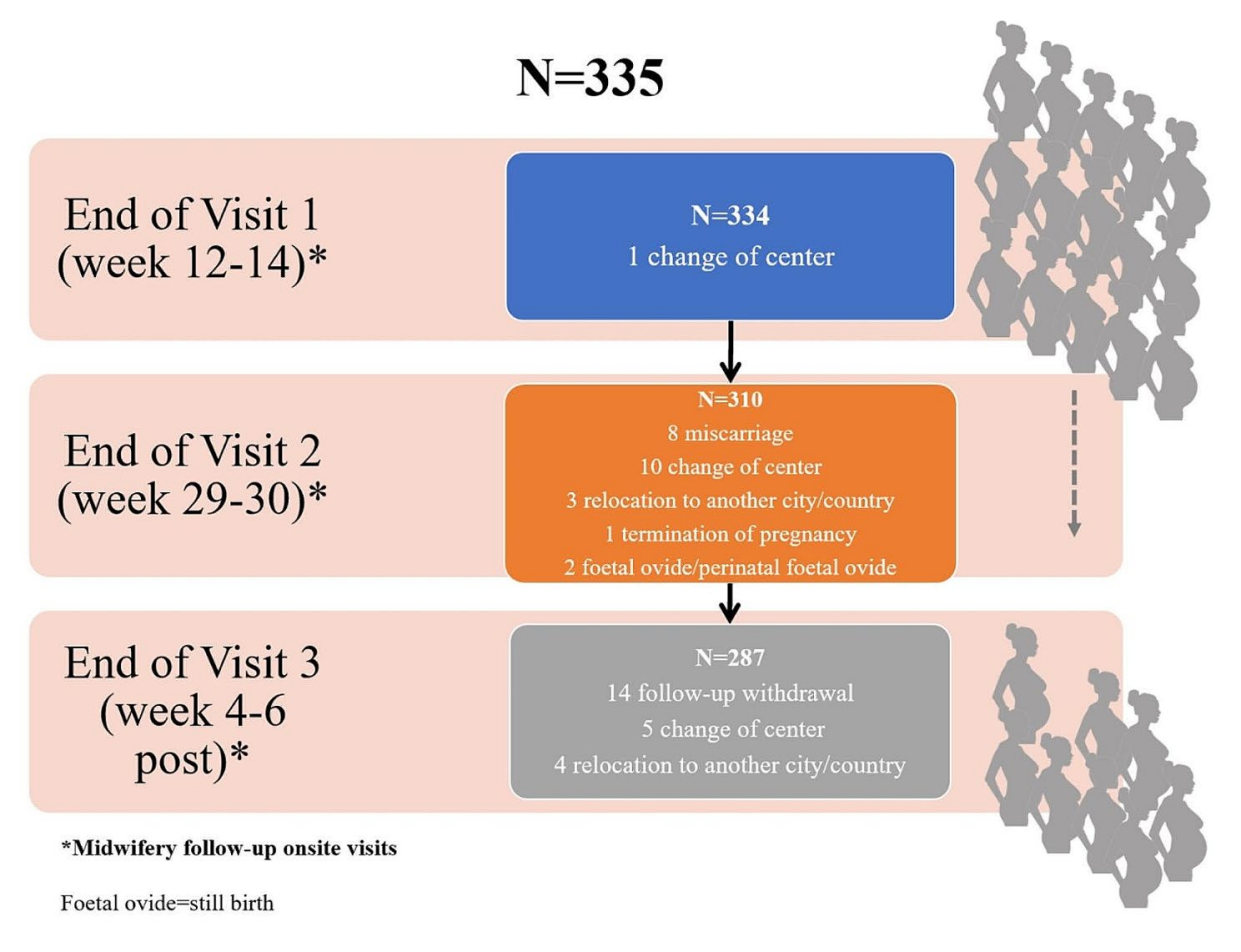
Flow diagram

A final sample of 335 pregnant women was initially included. Figure 2 shows losses across the study’s midwifery visits. Between visit 1 (weeks 12 and 14 of pregnancy) and visit 3 (weeks 4 and 6 postpartum) there was a sample loss of 14.2% (*N* = 48), of which 16.7% were miscarriages (in weeks 29–30 of pregnancy).

The majority of the sample either live with a partner or live with a partner and children (89.9%). 35% have a university education, while 19.8% have minimal or no education. 44.3% are skilled workers. Most report the couple’s income (92.8%). In relation to obstetric and clinical variables, the majority had planned the pregnancy (75.8%), 53.1% being first-time mothers. 8.1% had traumatic experiences related to a previous birth. 34.4% show current high/very high gestational risk level (Table 1). 6.6% (*N* = 22) of the sample suffered from maltreatment prior to the study, 2.4% (*N* = 8) from sexual abuse and 5.1% (*N* = 17) cited relationship problems with their partner.

A total of 14.6% (*N* = 49) of the women reported psychiatric disorders diagnosed in 1st-degree relatives, 9% (*N* = 30) showed severe mental illness in family members, and 2.1% (*N* = 7) reported cases of suicide in the family environment. 3% (*N* = 10) reported postpartum depression in mothers and/or sisters.

**Fig. 3:**
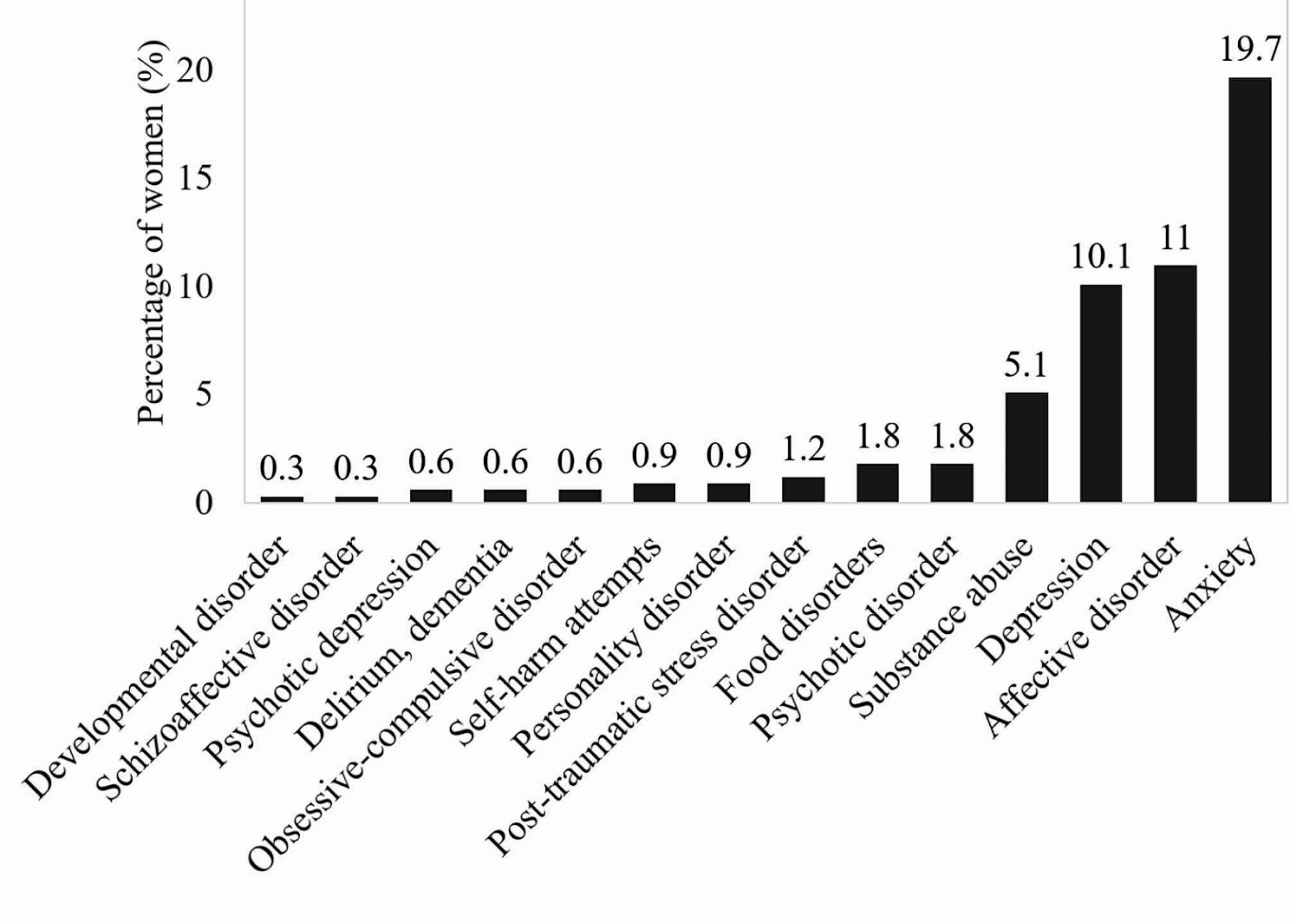
Psychiatric history of the participants

Figure 3 shows the psychiatric history of the sample. 18.5% (*N* = 62) of the women reported having received previous psychological/psychiatric treatment (7.5% [*N* = 25] psychopharmaceuticals, 5.4% [*N* = 18] psychological treatment, and 5.4% [*N* = 18] both types of approach), while 2.1% (*N* = 7) required hospitalisation for mental health problems (Table 1).

**Table 1:** Sociodemographic, clinical, and obstetric characteristics of the study sample at baseline (*N* = 335)

Sociodemographic variables	
Age	31 (6.0)
Cohabitation	
Lives with a partner	140 (41.8%)
Lives with a partner and children	161 (48.1%)
Lives with parents	11 (3.3%)
Lives with a partner and parents/in-laws	8 (2.4%)
Lives alone with children	2 (0.6%)
Lives with a partner and without her other children (does not have custody)	1 (0.3%)
Lives alone	1 (0.3%)
Other circumstances	11 (3.3%)
Country of origin	
Spain	229 (68.4%)
Other countries	106 (31.6%)
Level of education	
Illiterate/no studies/primary studies	66 (19.8%)
Secondary studies	151 (45.2%)
University studies	117 (35.0%)
Employment situation	
Skilled worker/administrative/manager or business owner	147 (44.3%)
Semi-skilled machine operator/unskilled worker	90 (27.1%)
Housewife	52 (15.7%)
Unemployed for the past 6 months and/or looking for work	38 (11.4%)
Student/unknown	5 (1.5%)
Partner’s income	310 (92.8%)
**Clinical and obstetric variables**	
Nulliparous	178 (53.1%)
Multiparous	157 (47.9%)
Previous foetal loss	2 (0.6%)
Previous traumatic birth experience	27 (8.1%)
Current planned pregnancy	254 (75.8%)
Current gestational risk level	
Low	144 (43.0%)
Medium	75 (22.4%)
High	92 (27.5%)
Very high	23 (6.9%)
Unknown	1 (0.3%)
Previous spontaneous/deferred abortions	79 (23.6%)
Voluntary maternal termination of pregnancy	46 (13.7%)
Termination of pregnancy for maternal or foetal medical reasons	8 (2.4%)
Previous caesarean	37 (11.0%)
Previous delivery with FK or vacuum	28 (8.4%)
Eutocic anterior delivery	102 (30.4%)
Previous high-risk pregnancy†	53 (15.8%)
Previous very high-risk pregnancy‡	13 (3.9%)
Current pregnancy through assisted reproduction	16 (4.8%)
In vitro fertilisation (IVF)	13 (3.9%)
Artificial insemination	3 (0.9%)
Smoker	39 (11.6%)
Number of cigarettes/day	6 (4.0)
Previous psychological/psychiatric treatments	62 (18.5%)
Hospitalisation for mental health problems	7 (2.1%)
Mistreated	22 (6.6%)
Sexual abuse	8 (2.4%)
Relationship problems with a partner	17 (5.1%)

Data presented as N (%) or mean (SD); FK: Forceps Kjelland

†The most common risk factor was gestational diabetes (2.1%, *N* = 7)

‡ The most common risk factor was pre-eclampsia (0.6%, *N* = 2)

### Clinical screening for detecting anxiety and depression

**Fig. 4:**
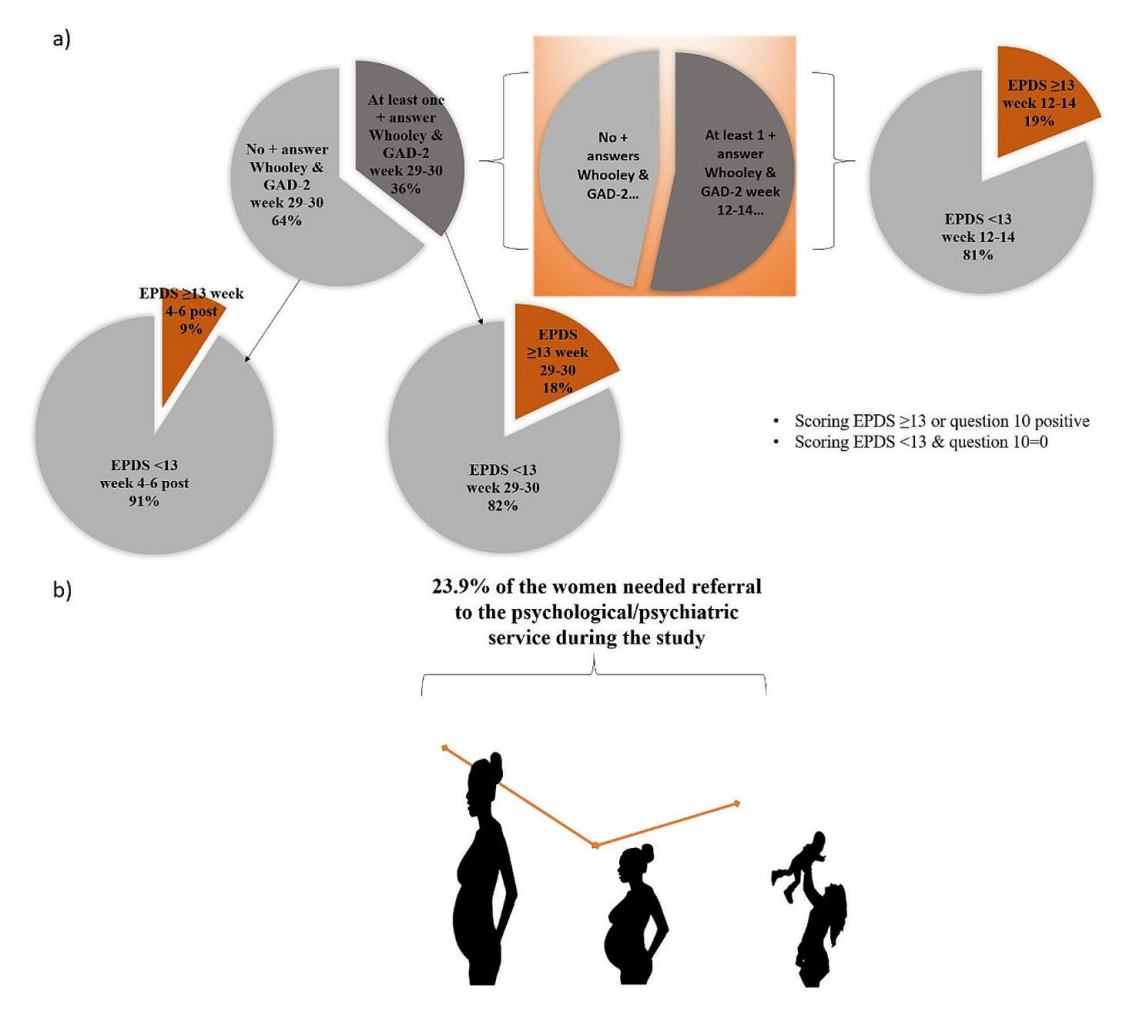
**(a)** Pie charts showing the evolution of cases with anxious and/or depressive symptomatology based on established screening. The percentages for each pie chart are calculated since the previous N from which they start (relative percentages). The EPDS scores threshold to consider pathology is established at 13 points and or to ask positively to question 10 (thoughts of harming oneself). In orange, women who over the course of the months of screening required a referral for diagnosis and assessment of psychological or psychiatric pathology. **(b)** Absolute percentages versus the original sample *N* = 335

At week 12–14 of pregnancy, 53.4% of the sample (*N* = 179) showed positive screening for symptoms of anxiety and depression (at least one positive response to the Whooley or the GAD-2 questions). Figure 4a) illustrates the evolution of cases showing anxious and/or depressive symptomatology as a function of the established screening. Figure 4b) shows specifically the percentages of women in the initial sample (*N* = 335) who scored ≥ 13 on the EPDS at the three midwife follow-up visits. Overall, there is a reduction in the percentage of cases scoring ≥ 13 on the EPDS across visits, which is most evident at the week 29–30 visit. A decrease of 2.3 is observed from week 12–14 antepartum to week 4–6 postpartum. A final sample of 80 out of 335 women (23.9%) were referred for psychiatric evaluation (in orange). See also Supplementary Fig. 1.

There were significant differences in the positive screening for anxiety and depression (including Whooley and GASD-2) between visit 1 and visit 2 (*p* < 0.001) with the proportion of positive cases at week 29–30 versus week 12–14 increased to 16.4%. The proportion of positive cases at weeks 12–14 that resulted in a negative screening at weeks 29–30 was 37.2%.

### Risk factors for positive screening (Whooley and GAD-2 questions)

#### Weeks 12–14 of pregnancy (visit 1)

Eleven sociodemographic, clinical, and obstetric variables were risk factors for positive screening at weeks 12–14.

### Sociodemographic variables

Age is lower in patients screened positive (mean [SD] of 30.4 [6.1] years versus 32.1 [5.7] years, *p* = 0.009). Smoking habit (smoking, but not the number of cigarettes), with women smokers having the highest percentages of positive screenings (15.1% [10.4%-20.9] versus 7.7% [4.3-12.7%], *p* = 0.035), education level, with illiterate women showing higher percentages of positive screenings (% [CI95%] 25.3% [19.3-32.0%] versus 13.5% [8.8-19.5%], *p* = 0.025), employment status, with women in skilled jobs having more negative screenings at week 12–14 than women in semi-skilled or unskilled jobs or unemployed (52.6% [44.7%-60.4] versus 37.1% [30.2-44.3%], *p* = 0.010).

A higher percentage of women living in a couple score < 13 on the scale (97.2% [93.6-99.1%] vs. 79.4% [63.8-90.3%], *p* < 0.001), and a higher percentage of women reporting a couple’s income score < 13 (93.8% [89.0-96.9%] vs. 82.4% [67.2-92.3%], *p* = 0.042).

### Clinical variables

Women with a history of previous depression have higher percentages of positive screenings (15.6% [10.9-21.5%] versus 3.8% [1.6-7.7%], *p* < 0.001), history of previous anxiety, with women with a history of anxiety having a higher percentages of positive screenings (27.9% [21.8-34.8%] versus 10.3% [6.2-15.74%], *p* < 0.001), previous psychological/psychiatric history, with women receiving previous treatment having higher percentages of positive screenings (25.7% [19.7-32.4%] versus 10.3% [6.2-15.7%], *p* < 0.001), type of treatment (psychological and/or pharmacological), with women who have received some treatment, especially pharmacological, having more cases of positive screening (cases with pharmacological treatment, 10.1% [6.3-15.1%] versus 4.5% [2.0-8.6%], *p* = 0.005), mistreated, with mistreated women having higher percentages of positive screenings (11.2% [7.2-16.4%] versus 1.3% [0.3-4.0%], *p* < 0.001), and marital problems, with women who are in a bad relationship having higher percentages of positive screenings (7.8% [4.6-12.4%] versus negative 1.9% [0.5-5.0%], *p* = 0.014). A higher percentage of women reporting cases of suicide in the family environment score ≥ 13 (0.7% [0.1-3.2%] vs. 8.8% [2.5-21.7%], *p* = 0.022), more women with previous depression history score ≥ 13 (10.3% [6.2-16.1%] vs. 38.2% [23.4-55.0%], *p* < 0.001), more women with previous anxiety history score ≥ 13 (58.8% [42.1-74.1%] vs. 20.7% [14.7-27.8%], *p* < 0.001), more non-mistreated women score < 13 (92.4% [87.3-95.9%] vs. 73.5% [57.2-86.0%], *p* = 0.004), and marital problems, with more women without a bad relationship scoring < 13 on the scale (95.9% [91.7-98.3%] vs. 76.5% [60.5-88.2%], *p* < 0.001).

### Obstetric variables

Only one variable emerged as an obstetric risk factor: voluntary termination of pregnancy, with women who voluntarily terminate pregnancy having more positive screenings than women who do not (17.9% [12.8-24.0%] versus 9.0% [5.2-14.2%], *p* = 0.010). A higher percentage of women with a current planned pregnancy score < 13 on the scale (75.9% [68.4-82.3%] vs. 55.9% [39.3-71.5%], *p* = 0.019).

### Weeks 29–30 of pregnancy (visit 2)

Ten sociodemographic, clinical, and obstetric variables were risk factors for positive screening responses at weeks 29–30.

### Sociodemographic variables

Educational level, with illiterate women having higher percentages of positive screenings (27.5% [19.8-36.4%] versus 15.6% [11.1-21.1%], *p* = 0.019). Type of job position affects EPDS score, i.e., a higher percentage of skilled workers score < 13 (40.4% [30.7-50.8%] vs. 20.0% [7.2-40.8%], *p* = 0.019).

### Clinical variables

Women with a history of depression show higher percentages of positive screenings (17.3% [11.1-25.1%] versus 7.0% [4.1-11.2%], *p* = 0.005), history of anxiety, with women with a history of previous anxiety showing higher percentages of positive screenings (35.5% [27.0%-44.7] versus 12.6% [8.5-17.7%], *p* < 0.001), prior psychological/psychiatric treatment, with women who received prior treatment having higher percentages of positive screenings (35.5% [27.0-44.7%] versus 10.1% [6.4-14.8%], *p* < 0.001), type of treatment (psychological and/or pharmacological), with women who have received some treatment having more cases of positive screening (cases with psychological treatment: 14.5% [8.9-22.0%] versus 1.0% [0.2-3.2%], *p* = 0 < 0.001), mistreated, with mistreated women having higher percentages of positive screenings (11.8% [6.8-18.8%] versus 3.0% [1.3-6.1%], *p* = 0.002), sexual abuse, with sexually abused women having higher percentages of positive screenings (5.5% [2.3-10.9%] versus 0.5% [0.1-2.3%], *p* = 0.009), and marital problems, with women who are in a bad relationship having higher percentages of positive screenings (10.0% [5.4-16.6%] versus 2.0% [0.7-4.7%], *p* = 0.003). More women without previous depression history score < 13 on the scale (89.0% [81.4-94.2%] vs. 55.0% [33.8-74.9%], *p* < 0.001), more women with previous anxiety history score ≥ 13 (55.0% [33.8-74.9%] vs. 30.8% [22.0-40.7%], *p* = 0.04), and marital problems, with more women without a bad relationship scoring < 13 (94.5% [88.4-97.9%] vs. 70.0% [48.3-86.4%], *p* = 0.004).

### Obstetric variables

Two obstetric variables emerged as risk factors associated with screening responses at 29–30 weeks: previous spontaneous/deferred miscarriages, with women with previous spontaneous/deferred miscarriages having more positive screenings than women without (33.6% [25.3-42.8%] versus 16.6% [11.9-22.2%], *p* < 0.001), the level of current gestational risk, with a trend to show more positive screening for those women at higher risk (37.3% [28.7-46.5%] versus 21.1% [15.9-27.2%], Fisher exact test, *p* = 0.003). There were no associated obstetric risk factors at visit 2.

### Week 4–6 postpartum (visit 3)

Eight sociodemographic, clinical and obstetric variables are risk factors for scoring ≥ 13 on the EPDS at week 4–6 postpartum.

### Sociodemographic variables

Age is higher in patients scoring ≥ 13 on the EPDS (35.0 [18.0–45.0] years vs. 32.0 [18.0–44.0] years, *p* = 0.035),

### Clinical variables

More women without previous depression history score < 13 (91.2% [87.2-94.2%] vs. 69.2% [50.2-84.2%], *p* < 0.001), more women without previous anxiety history score < 13 (81.9% [76.9-86.2%] vs. 50.0% [31.6-68.4%], *p* < 0.001), women without a previous psychological/psychiatric treatment score < 13 (83.5% [78.6-87.6%] vs. 50.0% [31.6-68.4%], *p* < 0.001), type of treatment (psychological and/or pharmacological), with women who have received both treatments scoring ≥ 13 (19.2 [7.7%-37.1] vs. women receiving only one type of treatment 4.2% [3.1-8.6%], *p* < 0.001), more non-mistreated women score < 13 (95.0% [91.8-97.2%] vs. 76.9% [58.5-89.7%], *p* = 0.004), and marital problems, with more women without a bad relationship scoring < 13 on the scale (97.3% [94.8-98.8%] vs. 73.1% [54.3-87.1%], *p* < 0.001).

### Obstetric variables

A higher percentage of women with a current planned pregnancy score < 13 (78.8% [73.6-83.5%] vs. 61.5% [42.4-78.2%], *p* = 0.045).

See Supplementary Fig. 2 for a mapping of all risk factors.

## Discussion

There is little evidence that screening to identify and treat depression during pregnancy improves outcomes. This may be due to variations in access to resources and appropriate treatment once a diagnosis of depression and/or anxiety has been established. However, screening for depression during pregnancy may provide some self-awareness of the risk of depression and anxiety. This study assesses for the first time the prevalence of anxiety and depression and their associated risk factors throughout the pregnancy and postpartum process in a large population of women attending the public health system, using a new screening tool for early detection of mental health problems.

### Clinical screening for detecting anxiety and depression

Around the first trimester of pregnancy, just over half of the sample (53%) showed positive initial screening for symptoms of anxiety and depression. Assessing the EPDS score ≥ 13 at this time, we found that 10% of the entire sample required referral to the psychological/psychiatric service during the study, with a reduction in the percentage of women scoring ≥ 13 on the EPDS during the period of the visits, this reduction being most evident at the 29–30-week visit. We note that the first part of the screening alone showed that half of the women were potentially at risk, while the EPDS assessment gives more accurate values (around 19%). A total of 80 women out of 335 (23.9%) were ultimately referred for psychiatric assessment throughout the study. Although anxiety and depression vary considerably depending on the population assessed [8, 9], this is certainly a high percentage, higher than previously reported in other studies [2, 3, 5–7]. One possible reason is that most of the previous literature does not pay attention to mental health screening in early pregnancy, starting to assess women’s mental health problems in their second trimester of pregnancy [3, 5] or as already as postpartum [2, 5, 7].

### Risk factors for positive anxiety and depression screening through Whooley and GAD-2 questions

There is a clear influence of a prior history of anxiety and depression on the initial positive screening, already in the first trimester of pregnancy and extending into the third trimester. Not only do the diagnoses of anxiety and depression tend to co-occur [33–35], but their risk factors appear to be similar. A history of anxiety or depression, or of previous psychiatric conditions, has been reported as a risk factor for anxiety and depressive symptoms not only in pregnant women [36] but also in other populations [37, 38]. So pregnant women with previous mental health conditions appear as a special target to be followed from the beginning of pregnancy.

We found smoking and the age to be risk factors for positive screening for anxiety and depression already in the first trimester. Increasing evidence suggests that smoking influences mental health negatively [39] and that there is a co-occurrence between depression/anxiety and smoking [40–42]. Although nicotine creates an instant feeling of relaxation, this feeling is momentary and soon gives way to withdrawal symptoms, increasing anxiety and tension. Nicotine stimulates the release of the chemical dopamine in the brain [43], which is initially involved in triggering positive feelings, but causes altered moods [44]. Reducing smoking rates in pregnancy is a priority [45]. Current evidence suggests that smoking is directly related to the onset of depressive and anxious symptoms early in pregnancy, so early action in this area appears to be of great benefit for mothers-to-be.

Adolescent mothers are known to experience health-related problems [46]. Younger age hinders economic and emotional stability for coping with financial, family, and social adversities. Younger mothers are more likely to be impoverished and to reside in socially and economically deprived families [47]. This can lead to anxiety and depression, especially during the postpartum period [48]. A very curious paradox appears in our data and that is that age appears as a differential risk factor pre- and postpartum, in an opposite way. We found that while in the early weeks of pregnancy, younger mothers are more likely to suffer anxiety and depression, in the postpartum period it is older mothers who show a greater tendency to depression. In the study of Agnafors et al. [48], only 3.5% of the sample are ≤ 20 years old, so the comparison with older mothers is quite unbalanced. Likewise, that study presents mothers with a mean age of 28 years (maximum range not shown). The mothers in that study are, on average, younger than the mothers included in our study, and their ages range from 18 to 45 years. Possibly the young mothers in that study had a specific casuistry that made them show more postpartum depression. In this sense, the characteristics of the mothers, their possibilities, coping skills, family and social support, and marital problems, among other factors, may come into play, as we shall discuss later.

Consistent with our postpartum findings, a previous study of pregnant women with a similar age range to those in our study (20–44 years) reported that older women had a higher risk of postpartum depression [49]. Possible reasons for this result could be multifactorial: the perception that older women find it more difficult to adapt to motherhood (because they have already had their lives structured for years, so there are difficulties in reconciling motherhood with work and personal life), and the lack of support from society due to prejudices in social norms around maternal age.

In this regard, we found that illiterate women had higher percentages of positive tests related to anxiety and depression. School dropout has been reported to be an even more influential factor in postpartum depressive symptoms than maternal age [47]. This factor appears to be influential throughout pregnancy from the beginning, so special attention should be paid to young and uneducated mothers in the first weeks of pregnancy.

Relationship problems (abuse and marital problems) seem to influence the likelihood of anxiety and depression in early pregnancy. The connection between any type of domestic violence and anxiety and depression is clear [50], affecting the mental health of pregnant women [51, 52]. It is essential to understand these aspects in the first weeks of pregnancy, to try to establish interventions aimed at minimizing these factors.

Difficulty in deciding whether to terminate a pregnancy voluntarily is an obvious cause of a mother’s anxiety and depression in early pregnancy, and it results in a risk of miscarriage in the third trimester. In both cases, it is a factor underlying the loss of the baby, but in early pregnancy, it is a personal decision, which increases the effect of regrets, etc., whereas in the third trimester, it is no longer voluntary, and the baby is lost for reasons other than personal decisions. Although depression has been found to occur up to eight times more frequently after childbirth than after miscarriage, it is also present in those cases [53]. Women who had a miscarriage scored higher on the anxiety and depression scale 10 days after miscarriage than the general population [53]. Although miscarriage is difficult to prevent in some cases, personal decisions about the termination of pregnancy can be worked on by professionals to reduce the impact of these decisions and offer support after the termination.

### Risk factors for positive results on the EDPS

The positive EDPS result shows a similar trend to that found in the initial Whooley and the GAD-2 screening, i.e., psychiatric history and a history of anxiety and depression are the risk factors that recur at each stage of pregnancy as predisposing to symptoms of anxiety and depression in the pregnant mother, and in the postpartum period, together with abuse and marital problems. The economic situation and the emotional stability of the couple are also important. Compared to married mothers, single mothers report being more likely to have experienced an episode of depression [54].

Current planned pregnancy is associated with lower rates of anxiety and depression in early pregnancy, consistent with a previous study [55]. Pregnancy intention is a complex concept involving different psychological aspects (e.g., psychological adjustment to the presence of a future baby, willingness to change one’s current life with the presence of another family member). This psychological preparation is essential to minimize the impact of stress and depression processes. We found that family suicide may increase the risk of psychiatric problems in pregnant women. Strategies learned in the family environment for coping and for solving problems seem to be important in reducing psychiatric problems.

It may therefore no longer be a question of screening for possible depressive states, but of providing general coping strategies for the postpartum period in the early stages of pregnancy as a vital element, and not simply focusing on childcare responsibilities alone [56].

#### Limitations of the study

This study is not without limitations. First, although the sample is very large, its representativeness should be mentioned. Adolescent women are not represented in the current sample. Existing studies suggest that pregnant adolescents are at greater risk of suffering from depressive symptoms than pregnant and postpartum adult women [57–59]. Our percentages should therefore be contextualized considering the age of the sample, because the percentages may have been higher if adolescent mothers, with possible different risk factors for psychiatric symptoms, had been included. Also, women who are followed up in the private healthcare system are not part of the recruited sample. Significant differences have been reported in obstetric intervention rates between those with private and public health coverage [60–62]. It is well-known that private sector healthcare facilities are associated with a substantial increase in caesarean deliveries worldwide [63–65], even among low-risk obstetric births [61, 66], and this increase may also be related to anxiety and depression. Anxiety felt by women before caesarean section may cause psychological problems: most women are afraid of facing this procedure that may pose unnecessary risks for them, which is very significantly related to the level of stress or anxiety of patients [67]; also, prolonged anxiety and depression have been observed after this medical intervention [68]. However, this study aimed to evaluate a standardised protocol recommended by the public health system to assess how this new screening can assist in the early detection of psychological symptoms, thereby reducing the economic and clinical-staffing burden of monitoring established psychiatric disorders at later stages. Secondly, considering that religious beliefs appears as a powerful coping strategy to help deal with stressful situations [69], and that religion has been related to different coping strategies for pregnancy-related processes that dismisse psychological and psychiatric symptoms [70, 71], our study has not assessed whether this factor is influencing our results. Future studies should take this factor into account. Thirdly, the study did not include variables related to the time of delivery (e.g., type of delivery, complications, etc.), and perhaps these variables could affect the EPDS postpartum scores.

One of the strengths of this study is that, although there were sample losses, given the long period over which these women were being followed, the sample reduction was not high.

## Conclusion

Although the existence of different antenatal, perinatal, and postnatal depression and anxiety risk factors in this cohort is of concern, screening in the first trimester of pregnancy may improve the mental health status of pregnant women, as well as reduce the subsequent burden in the mental health system. Care for young mothers in the early weeks and for older mothers during the postpartum period seems to be crucial.

The schedule of visits and follow-ups for pregnant women is likely to differ in the public health system from what is usually done in private practices, and a quick screening seems to be very useful to detect women potentially targeted for referral for diagnosis and assessment of psychological/psychiatric pathology in early stages, to catch them in time and prevent the disorder from becoming chronic, which would involve more expense and more effort from medical staff.

The fact that this protocol allows early psychological and psychiatric screening of pregnant women through the midwife is very valuable, as it reduces the burden of the medical staff who carry out the psychological assessments.

### Electronic supplementary material

Below is the link to the electronic supplementary material.

Supplementary Material 1

## Data Availability

The datasets and materials utilized in this study are available upon reasonable request from the corresponding author. Access to these resources is subject to ethical and legal constraints, and data-sharing will be in compliance with institutional and regulatory guidelines. Researchers interested in obtaining the data and materials should contact the corresponding author to initiate the request process. Please provide a detailed rationale for the request, and we will strive to facilitate access following applicable policies. For proprietary or confidential information that cannot be shared, summaries or excerpts that maintain the integrity of the research outcomes may be provided upon request. The study was conducted under ethical approvals and data protection regulations, and any data-sharing will be carried out while upholding participant confidentiality and data privacy.
