# Supplementary material for: Prevalence of anxiety and depression and their associated risk factors throughout pregnancy and postpartum: a prospective cross-sectional descriptive multicentred study
Source: BMC Pregnancy Childbirth. 2024 Jul 25;24:500. doi: 10.1186/s12884-024-06695-6 (PMC11270936; doi:10.1186/s12884-024-06695-6)
Supplement: Supplementary file 1 — Supplementary Material 1 [file 12884_2024_6695_MOESM1_ESM.docx]

Supplementary Material

**Sociodemographic and clinical screening for mental health disorders**

*Clinical screening for detecting anxiety and depression*

*The Whooley Questions*

The Whooley Questions (Whooley et al., 1997) are already recommended by NICE guidelines to identify people who may be at risk of depression. The two questions are: in the past month, a) have you felt down or depressed or hopeless? and b) have you been bothered by little interest or pleasure in doing things? Both items have a dichotomic answer (yes/no). A positive answer to one or both questions implies that the person should be better evaluated with a more thorough interview to diagnose the condition. This instrument was passed at 12-14 weeks of pregnancy and, if necessary, at 29-30 weeks of pregnancy.

*Generalised Anxiety Disorder Scale (GAD-2)*

The GAD-2 is a very brief and easy 2-item instrument to perform initial screening tool for generalized anxiety disorder (Kroenke et al., 2007). The GAD-2 is a version of the 7-item scale (GAD-7) (Spitzer et al., 2006) that incorporates the two first questions of the GAD-7. Specifically, the GAD-2 ask for over the last two weeks how often the women have been bothered by the following two problems: a) feeling nervous, anxious or on edge, and b) not being able to stop or control worrying. This instrument was passed at 12-14 weeks of pregnancy, at 29-30 weeks of pregnancy, and at 4-6 weeks postpartum according to Whooley’s results. This instrument was passed at 12-14 weeks of pregnancy and at 29-30 weeks of pregnancy.

*Edinburg Postnatal Depression Scale (EPDS)*

The EPDS is a 10-items self-report scale created to detect postnatal depression. This scale was initially developed to assist primary care health professionals in detecting mothers suffering from postpartum depression (Cox et al., 1987). It consists of 10 questions with four possible answers each (scored from 0 to 3, according to increased severity of the symptoms, except for three items, with reverse scored). The mother is asked to underline the response that comes closest to how she has felt during the previous seven days. All 10 short-statements must be completed. The time to complete the scale does not usually exceed 5 minutes. Scores ranges from 0 to 30. This tool has obtained satisfactory psychometric properties in the Spanish validation used in the current study, with a sensitivity of 79%, specificity of 95.5%, and with a positive predictive value of 63.2% and a negative predictive value of 97.7% (Garcia-Esteve et al., 2003). Following the NICE guidelines and the Pregnancy Control Protocol in Catalonia (Agència de Salut Pública de Catalunya) (Generalitat de Catalunya, 2018), a cut-off of ≥13 was used to consider the participant had a picture compatible with a depressive illness, to be referred for psychological and/or psychiatric diagnosis. The scale will not detect mothers with anxiety neuroses, phobias or personality disorders. This instrument was passed at 12-14 weeks of pregnancy, at 29-30 weeks of pregnancy, and at 4-6 weeks postpartum according to Whooley’s results.

**Discussion**

*Covid-19 impact*

This study is not exempt from considering mental health consequences of COVID-19 during the study period. There is a clear association between the occurrence of psychiatric disorders, including anxiety and depression, and the presence of COVID-19 (Taquet et al., 2021). Elevated symptoms of anxiety and depression among different pregnant populations during the ongoing COVID-19 outbreak (Lebel et al., 2020; Preis et al., 2020; Saccone et al., 2020; Wu et al., 2020; Xu et al., 2021) have been reported. A recent cross-sectional survey in pregnant and postpartum women across 64 countries (https://corona.pregistry.com/) elevated posttraumatic stress, anxiety/depression, and loneliness have been shown to be highly prevalent in this population during the COVID-19 pandemic (Basu et al., 2021). Public health interventions need to explicitly address the impact of COVID-19 related stressors on mental health in perinatal women, as prevention of viral contact itself does not alleviate the impart of the pandemic in mental health.

*Implications for practice and future studies*

Little research has examined the feasibility and utility of screening for prenatal stress. Clinical screening for depression or anxiety in prenatal and postpartum healthcare has been widely recommended but was also potentially challenging., i.e. because of the tools to use or the need for clinicians to follow-up pregnant women. The current novel screening protocol for anxiety and depression in pregnant women, allows for a very quick and fast initial screening by the midwifery, which reduces costs, time and resources.

We found that more than 50% of the studied women here were potentially in risk for anxiety and depression. This percentage may seem really high, resulting in initial high rates of false-positive results, however when passing the EPDS after this step, the percentage remains at 19% of the sample, which is very reasonable. Low rates of clinical follow-up and referral, and subsequent lack of follow-up and metal treatment, are worse than preliminary screening with proven false positives. So, our results reflect the need for future early preventive perinatal mental health screening, already in the first weeks of pregnancy, to ensure optimal health outcomes for both mother and baby, as well as for the rest of the family. I.e., it has been reported that antenatal mindfulness intervention in pregnant women at early stages reduces anxiety and depression (Woolhouse et al., 2014).

**Supplementary Figures**

Supplementary Figure 1. Screening results for anxiety and depression symptoms in pregnant women and during the early postpartum period by the Protocol for Pregnancy Control (2018) (Generalitat de Catalunya, 2018).


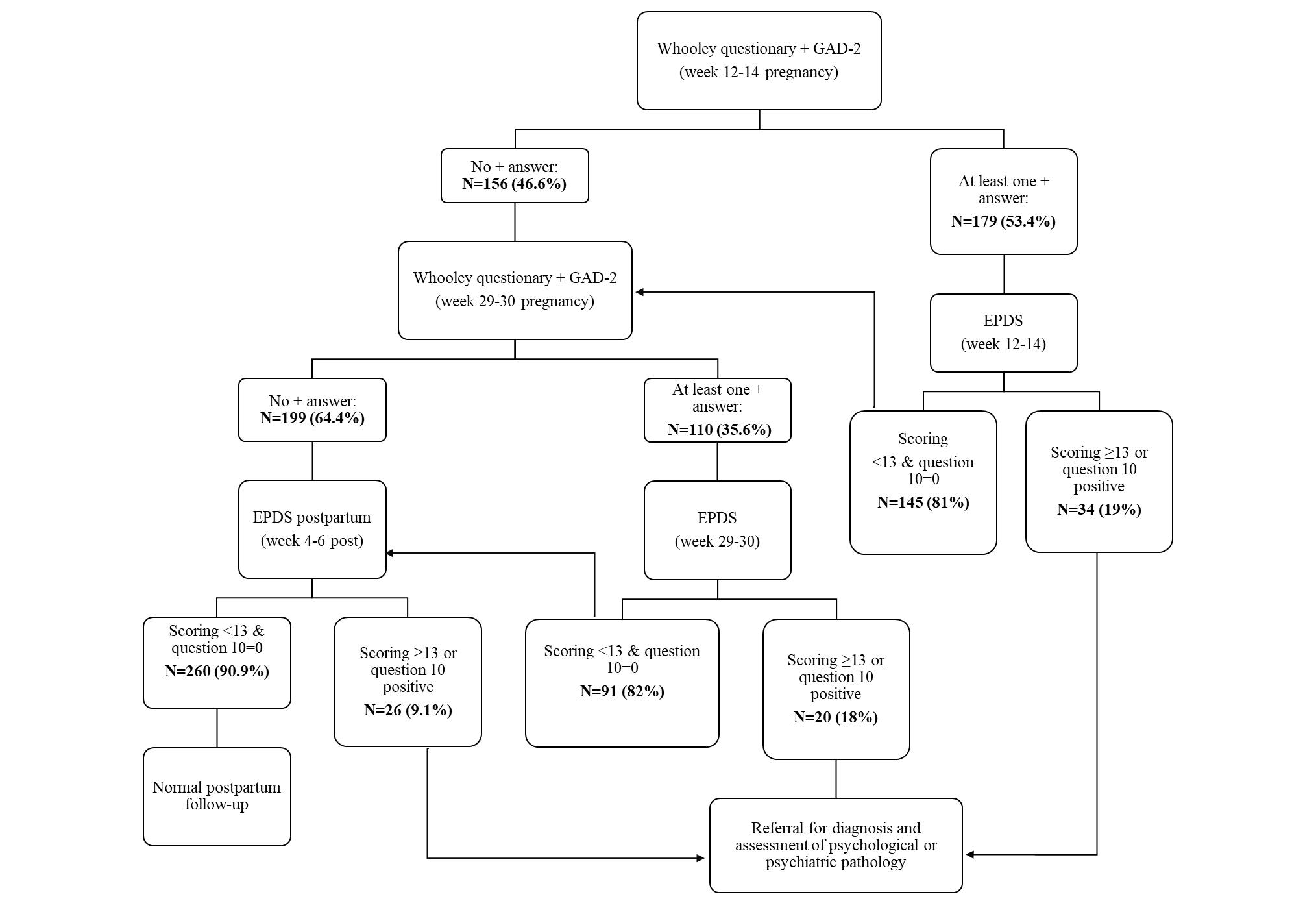


Relative percentages calculated on the basis of the previous N in the chain of events

Supplementary Figure 2. Risk factors for positive screening at each stage of the protocol.


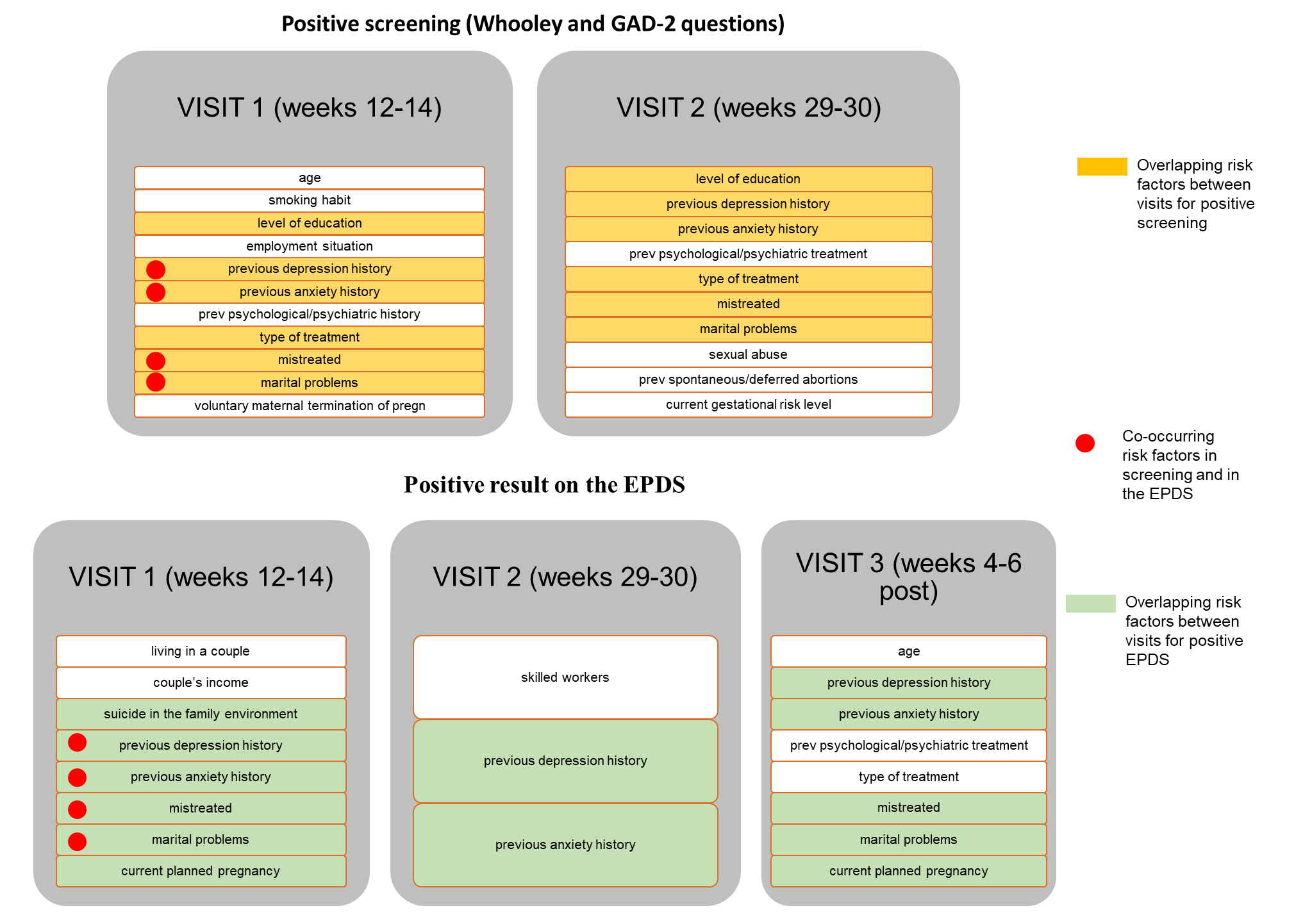


**References**

Basu, A., Kim, H.H., Basaldua, R., Choi, K.W., Charron, L., Kelsall, N., Hernandez-Diaz, S., Wyszynski, D.F., Koenen, K.C., 2021. A cross-national study of factors associated with women’s perinatal mental health and wellbeing during the COVID-19 pandemic. PLOS ONE 16, e0249780. https://doi.org/10.1371/journal.pone.0249780

Generalitat de Catalunya, Departament de Salut, 2018. Protocol de seguiment de l’embaràs a Catalunya. https://www.bio-centre.ad/Uploads/Noticies/protocol-seguiment-embaras-2018_def.pdf

Lebel, C., MacKinnon, A., Bagshawe, M., Tomfohr-Madsen, L., Giesbrecht, G., 2020. Elevated depression and anxiety symptoms among pregnant individuals during the COVID-19 pandemic. J Affect Disord 277, 5–13. https://doi.org/10.1016/j.jad.2020.07.126

Preis, H., Mahaffey, B., Heiselman, C., Lobel, M., 2020. Vulnerability and resilience to pandemic-related stress among U.S. women pregnant at the start of the COVID-19 pandemic. Social Science & Medicine 266, 113348. https://doi.org/10.1016/j.socscimed.2020.113348

Saccone, G., Florio, A., Aiello, F., Venturella, R., Angelis, M.C.D., Locci, M., Bifulco, G., Zullo, F., Sardo, A.D.S., 2020. Psychological impact of coronavirus disease 2019 in pregnant women. American Journal of Obstetrics & Gynecology 223, 293–295. https://doi.org/10.1016/j.ajog.2020.05.003

Taquet, M., Luciano, S., Geddes, J.R., Harrison, P.J., 2021. Bidirectional associations between COVID-19 and psychiatric disorder: retrospective cohort studies of 62 354 COVID-19 cases in the USA. The Lancet Psychiatry 8, 130–140. https://doi.org/10.1016/S2215-0366(20)30462-4

Woolhouse, H., Mercuri, K., Judd, F., Brown, S.J., 2014. Antenatal mindfulness intervention to reduce depression, anxiety and stress: a pilot randomised controlled trial of the MindBabyBody program in an Australian tertiary maternity hospital. BMC Pregnancy Childbirth 14, 369. https://doi.org/10.1186/s12884-014-0369-z

Wu, Y., Zhang, C., Liu, H., Duan, C., Li, C., Fan, J., Li, Hong, Chen, L., Xu, H., Li, Xiangjuan, Guo, Y., Wang, Y., Li, Xiufeng, Li, J., Zhang, T., You, Y., Li, Hongmei, Yang, S., Tao, X., Xu, Y., Lao, H., Wen, M., Zhou, Y., Wang, J., Chen, Y., Meng, D., Zhai, J., Ye, Y., Zhong, Q., Yang, X., Zhang, D., Zhang, J., Wu, X., Chen, W., Dennis, C.-L., Huang, H., 2020. Perinatal depressive and anxiety symptoms of pregnant women during the coronavirus disease 2019 outbreak in China. American Journal of Obstetrics & Gynecology 223, 240.e1-240.e9. https://doi.org/10.1016/j.ajog.2020.05.009

Xu, K., Zhang, Ya, Zhang, Yuanyuan, Xu, Q., Lv, L., Zhang, J., 2021. Mental health among pregnant women under public health interventions during COVID-19 outbreak in Wuhan, China. Psychiatry Res 301, 113977. https://doi.org/10.1016/j.psychres.2021.113977
